# Van der Waals superlattices

**DOI:** 10.1093/nsr/nwab166

**Published:** 2021-09-02

**Authors:** Huaying Ren, Zhong Wan, Xiangfeng Duan

**Affiliations:** Department of Chemistry and Biochemistry, University of California, Los Angeles, USA and; Department of Chemistry and Biochemistry, University of California, Los Angeles, USA and; Department of Chemistry and Biochemistry, University of California, Los Angeles, USA and; California NanoSystems Institute, University of California, Los Angeles, USA

## Abstract

This perspective explores the development of van der Waals superlattices, which are manipulated and constructed at atomic thick level, and points out potential applications and possible future directions of this new class of materials.

Semiconductor technology has enabled many essential devices, including transistors for computing and communication, diodes for solid-state lighting and photovoltaics. Behind all these devices, the material foundation is a series of highly elaborated heterostructures and superlattices [1]. To this end, integrating different materials into heterostructures or superlattices with well-defined spatial modulation of chemical compositions and electronic structures is central for generating designed electronic functions and has been a continued pursuit of the materials science community. The traditional approaches to heterostructures and superlattices, such as epitaxial growth, usually rely on the atom-to-atom covalent bonds to join the constituent materials and are often limited by strict lattice matching or processing compatibility requirements. High-quality heterostructures or superlattices can only be obtained between materials with nearly identical lattice structures and thus very similar electronic properties (e.g. Ga_1−x_Al_x_As with slightly different compositions) [2]. A slight mismatch would inevitably lead to interfacial defects/strains, which could often propagate well beyond the interface to form extensive dislocations in bulk lattices, and in some cases, totally disordered interfacial layers (Fig. 1a and b) [3]. As a result, materials with substantially different lattice structures can hardly be integrated together without generating too many defects that may fundamentally alter their electronic functions.

**Figure 1.**
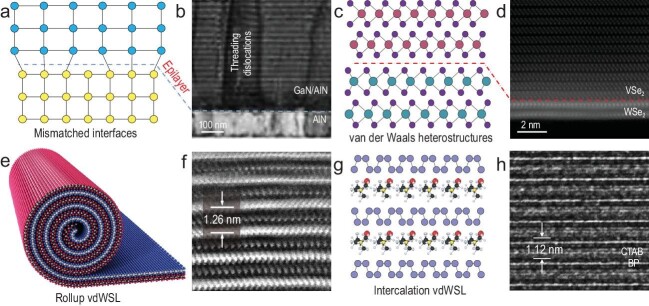
Schematic illustrations and cross-sectional electron microscopic structures of conventional bonded superlattices, vdWHs and vdWSLs. (a) Conventional bonded interfaces with a lattice mismatched interface. (b) Cross-sectional images of a (GaN/AlN) superlattice epitaxially grown on AlN/Si substrate. Threading dislocations are clearly observed across the entire superlattice stack due to lattice mismatch [3]. The dark cyan dotted line on (a) and (b) indicates the epilayer interfaces. (c) Bonding-free vdW interface of vdWHs. (d) Cross-sectional image of a VSe_2_/WSe_2_ vdWH [6]. The red dotted line on (c) and (d) indicates the vdWH interfaces. (e and f) Schematic illustration and cross-sectional image of a SnS_2_/WSe_2_ rolled-up vdWSL [8]. (g and h) Cross-sectional illustration and image of monolayer phosphorene molecular superlattices [11]. Adapted from refs [3,6,8,11] with permissions.

The emergence of two-dimensional atomic crystals (2DACs) has inspired new thinking on heterostructure/superlattice construction. These 2DACs consist of a large family of layered materials with covalently bonded atomic layers weakly held together by van der Waals (vdW) interactions, and can be readily isolated into single- or few-atom thick nanosheets. Different atomic layers can be mixed and reassembled together to form a new generation of artificial heterostructures [4]. Without any interfacial chemical bond between neighboring layers, the 2D atomic layers are held together by vdW interactions, and therefore are often called vdW heterostructures (vdWHs). Similarly, a periodic repeat of such vdWHs can lead to the formation of high-order vdW superlattices (vdWSLs). The bond-free vdW interaction between the neighboring layers offers a natural mechanism for interfacial strain relaxation, allowing a nearly perfect crystalline structure within each constituent layer to be retained regardless of the structural differences between them (Fig. 1c), which is in stark contrast to the conventional epitaxial superlattices that can only form between alike lattices (Fig. 1a and b). Such a bond-free vdW integration enables the creation of high-quality vdWSLs from many 2D crystals with very different lattice structures or physical properties beyond the limits of lattice matching requirements, and in principle provides vast flexibility for creating an entirely new generation of artificial vdWSLs with designable structural and electronic properties, enabling novel applications beyond the reach of traditional superlattices.

To date, the 2D vdWHs and vdWSLs are mostly obtained through a mechanical exfoliation and arduous layer-by-layer restacking process. This approach is highly versatile for creating diverse vdWHs, but typically has rather limited yield or throughput [1,5]. Alternatively, the chemical vapor deposition (CVD) approach has also been used for directly synthesizing 2D vdWHs (Fig. 1d) [6,7], but is often limited to low-order structures with only two, or few, distinct blocks. In general, the preparation of high-order vdWSLs with an increasing number of alternating units requires repeated restacking or synthesis steps, which is exponentially more challenging due to the limited yield and/or material damages associated with each sequential step. Consequently, the vdWHs/vdWSLs explored to date are usually limited to relatively simple heterostructures with only a small number of blocks.

Most recently, a unique capillary-force-driven rolling-up strategy was used to produce synthetic high-ordered SnS_2_/WSe_2_ vdWSLs with alternating monolayers of WSe_2_ and SnS_2_ (Fig. 1e and f) [8]. Importantly, this approach is rather general and can be readily extended for preparing a wide range of vdWSLs consisting of two, three or more types of 2D atomic layers. This rollup strategy provides a straightforward approach for producing high-order vdWSLs with deliberately designed atomic-scale integration of distinct atomic layers, electronic structures and physical properties (e.g. semiconductor, superconductor, metal, Weyl semimetal, topological insulator and ferromagnet). This allows vast freedom to tailor the electronic band modulation, interlayer coupling, chirality and topology of the resulting vdWSLs by design.

Alternatively, high-order vdWSLs may also be constructed through a unique intercalation process. The non-bonding nature between neighboring atomic layers in the 2DACs features a unique vdW gap, into which a variety of foreign species could be inserted without breaking the in-plane covalent bonds to create high-order vdWSLs. Such an intercalation process allows for inserting diverse foreign species, such as atoms, ions and molecules, into a wide range of 2D layered host crystals, creating a new family of high-order intercalation superlattices consisting of alternating layers of the covalently bonded atomic layers and the self-assembled layers of the intercalants [9,10]. By varying the host 2DACs and tailoring the composition, size, structure and electronic properties of the intercalants, an expansive family of high-order intercalation superlattices may be produced with widely variable compositional/structural features and tunable physical/chemical properties.

Although intercalation approaches could in principle provide a great deal of flexibility for integrating different atomic or molecular species into a superlattice structure with atomic precision, current studies are largely limited to highly reactive species (e.g. lithium) that can fundamentally alter the structural and electronic properties of the hosting 2DACs, or passive molecules (e.g. fully saturated insulating molecules) that show little electronic interaction with the hosting 2DACs (Fig. 1g and h) [11]. In either case, the intercalated layers do not directly show optical, electronic or magnetic functions or exhibit active electronic coupling with the hosting atomic layers, and thus can be largely viewed as ‘passive intercalation superlattices’, in which the intercalated layers only serve as passive spacers to expand the interlayer separation or decouple the electronic coupling between the original hosting layers.

To move a step further, a more interesting class of high-order superlattices should consist of both the functional hosting atomic layers (e.g. semiconductors such as MoS_2_ and WSe_2_) and functional intercalant layers (e.g. organic semiconductors with proper HOMO/LUMO positions and magnetic coordination complexes) [9]. In particular, by properly controlling the electronic band offset and the intricate electronic interactions between the hosting layers and the intercalant layers, it is possible to create a new generation of ‘functional intercalation vdWSLs’ with tunable electronic, optical and magnetic properties through interlayer proximity effect between the host and the intercalant layers. The flexible combination of diverse 2DACs with widely variable functional intercalants can result in the freedom to modulate the electronic or topological nature of the energy bands of the resulting superlattices, offering exciting opportunities for controlling the generation, confinement and modulation of charge, exciton and spin states at the limit of single-atom thickness and across the alternating layers.

However, it is non-trivial to intercalate the electronically active functional intercalants into 2DACs and create ‘functional intercalation vdWSLs’. The intercalation of functional molecules is fundamentally more challenging than the passive molecules explored to date [9]. First, functional species with intermediate HOMO/LUMO levels are usually not active enough to spontaneously react with and intercalate into typical semiconducting 2DACs through a direct charge transfer process (e.g. similar to Li intercalation). Second, such functional species usually show poor electrochemical stability, which limits the applicable electrochemical window for electrochemical intercalation. Thus, the synthetic challenge remains a major bottleneck for functional molecule intercalation in 2DACs from both the fundamental material chemistry and potential technological application points of view. Recently, an exfoliation and reassembly approach was reported for preparing similar 2D/molecular vdWSLs [12]. Without an aggressive electrochemical process, this approach may offer an alternative mild strategy for creating functional intercalation superlattices. Additionally, the recently emerged 2D organic/inorganic hybrid perovskites share a similar alternating layered structure between inorganic layers and organic layers, and may be viewed as a special class of vdWSLs [13], and could be prepared through the direct synthetic pathway.

The bond-free vdWSLs bring an unprecedented degree of freedom for atomic-scale integration of highly distinct atomic or molecular layers with radically different chemical compositions, distinct structural configurations and widely tunable optical, electronic, spintronic and magnetic properties (Fig. 2) [10,14–16]. It breaks the limits set by the lattice matching or processing compatibility requirements for traditional epitaxial superlattices and enables a totally new class of artificial superlattices with widely tunable atomic structure, band offset, interlayer coupling, dimensionality, chirality and topology. This distinct flexibility makes vdWSLs a rich material platform for exploring intricate electronic and spin interactions between the monolayer atomic crystals and/or sandwiched molecular layers, and for fundamental studies of electrochemistry, ferroelectricity, ferromagnetism, optoelectronics and superconductivity at such atomic interfaces. It may seed transformative advances across diverse areas ranging from traditional electronics,

 

optoelectronics and electrochemistry to the emerging areas of spintronics and quantum information science. Considering the rich electronic functions generated from a few limited semiconductor heterostructures and superlattices in today's semiconductor technology, the opportunities that may be brought by such highly versatile vdWSLs with nearly infinite variations seem boundless. It could define a new paradigm of artificial materials for new physics, new devices and new technologies.

**Figure 2.**
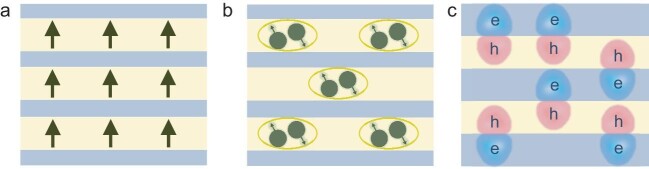
Schematic illustrations of modulating magnetic, electronic and optical properties in functional vdWSLs. (a) Schematic drawing of an artificial superlattice with functional layer consisting of ferromagnet and semiconductor layers. In this way, electronic control of ferromagnet ordering through interlayer coupling could be achieved. (b) Schematic drawing of an artificial superlattice with superconductor/semiconductor layers. In such ways, Cooper pairs can be injected into a high mobility semiconductor through proximity effects. (c) Schematic drawing of an artificial superlattice with different electron and hole affinity to realize interlayer exciton or electron-hole superfluid in bulk superlattices.
